# The edge-to-edge repair of the iatrogenic torrential tricuspid regurgitation complicating transvenous pacemaker lead extraction: a case series

**DOI:** 10.1093/ehjcr/ytag164

**Published:** 2026-03-06

**Authors:** Jarosław Skowroński, Adam Rdzanek, Patrycjusz Stokłosa, Krzysztof Jaworski, Piotr Scisło, Agnieszka Kapłon-Cieślicka, Joanna Zakrzewska, Jerzy Pręgowski

**Affiliations:** National Institute of Cardiology, ul. Alpejska 42, 04-628 Warsaw, Poland; 1st Chair and Department of Cardiology, Medical University of Warsaw, ul. Banacha 1A, 02-091 Warsaw, Poland; National Institute of Cardiology, ul. Alpejska 42, 04-628 Warsaw, Poland; National Institute of Cardiology, ul. Alpejska 42, 04-628 Warsaw, Poland; 1st Chair and Department of Cardiology, Medical University of Warsaw, ul. Banacha 1A, 02-091 Warsaw, Poland; 1st Chair and Department of Cardiology, Medical University of Warsaw, ul. Banacha 1A, 02-091 Warsaw, Poland; National Institute of Cardiology, ul. Alpejska 42, 04-628 Warsaw, Poland; National Institute of Cardiology, ul. Alpejska 42, 04-628 Warsaw, Poland

**Keywords:** tricuspid regurgitation, Pacemaker lead extraction, Transcatheter edge-to-edge repair, Case series

## Abstract

**Background:**

New tricuspid regurgitation (TR) or worsening of baseline pathology may appear after transvenous pacemaker lead extraction (TLE). In case of severe TR induced by TLE, surgical correction might be required. However, the patients in whom TLE is performed are not always appropriate candidates for open-heart intervention.

**Case Summary:**

Herein, we present four patients with iatrogenic TR significantly increased to severe-torrential grade after TLE, who were disqualified from surgery. In three cases, the regurgitation was associated with an avulsion of the papillary muscles or chordae tendineae. In one case, it was likely due to the perforation of the tricuspid leaflet during TLE. Patients were treated with transcatheter tricuspid edge-to-edge repair (T-TEER), in three cases successfully.

**Discussion:**

Percutaneous repair of TLE-related TR is feasible and may be a valuable therapeutic option in patients with prohibitive surgical risk. One of the most important factors leading to a T-TEER procedural success is the accurate assessment of the tricuspid regurgitation mechanism. In the fourth case, identification of the underlying regurgitation cause was limited by the image quality in the transoesophageal examination.

Learning points

- Percutaneous repair of TLE-related tricuspid regurgitation might be a valuable therapeutic option, especially in patients who are not candidates for surgical intervention.
- Most important factors leading to a T-TEER procedural success are accurate assessment of the tricuspid regurgitation mechanism.
- Tricuspid regurgitation following TLE can result in serious clinical consequences, including rapidly progressing heart failure with limited response to medical therapy.

## Introduction

**Figure 1:**
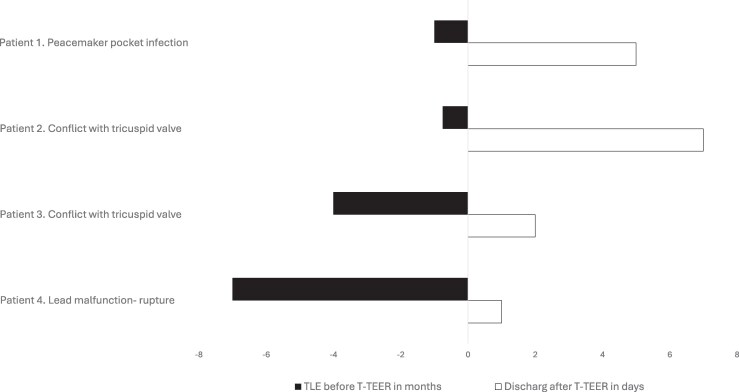
Clinical timeline. Time from TLE to TEER is presented in months; time from TEER to hospital discharge in days.

Severe tricuspid regurgitation (TR) is present in up to 40% of patients who received an implantable cardioverter-defibrillator or a pacemaker within 5 years after the procedure.^1^ This may be caused by lead impingement on the leaflets or chordae tendineae/papillary muscle injury, which resembles a primary valvular pathology.^2^ Other potential causes include tricuspid annular dilatation and/or right ventricular remodelling.^2^ New TR or worsening of baseline pathology may also appear after transvenous pacemaker lead extraction (TLE).^3^ In case of severe TR induced by TLE, surgical correction might be required.^4^ However, the patients in whom TLE is performed are not always appropriate candidates for open-heart intervention. Herein, we present four patients with iatrogenic severe-to-torrential TR secondary to TLE who were disqualified from surgery and underwent tricuspid transcatheter edge-to-edge repair (T-TEER), in three cases successful. *Figure 1*.

## Patient 1

**Figure 2:**
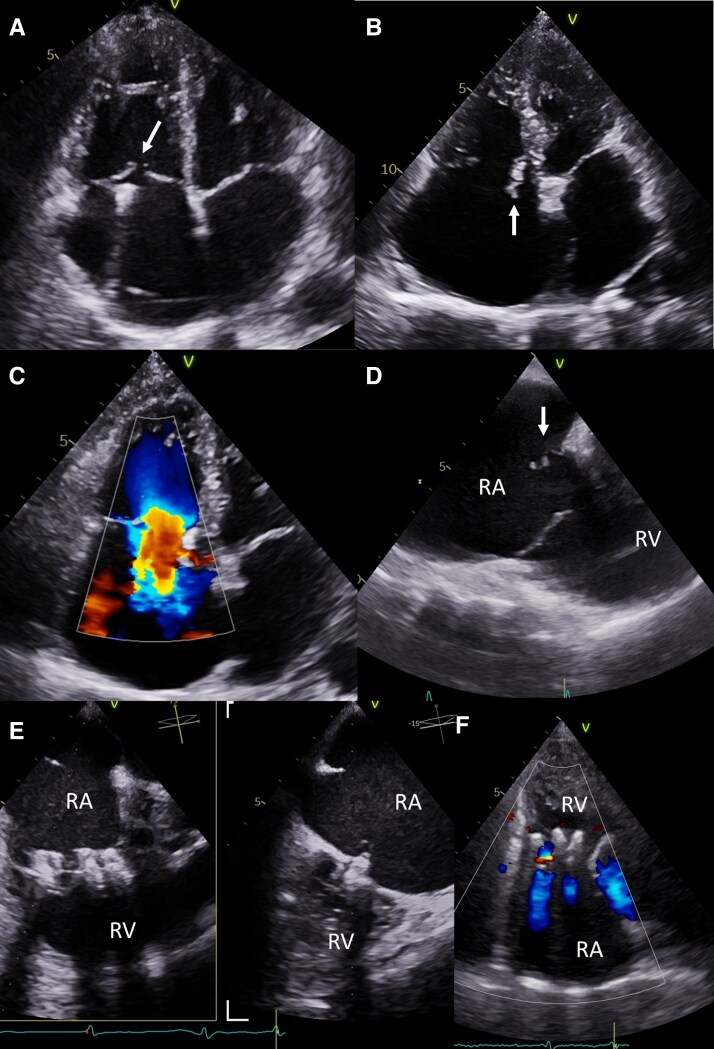
Patient 1 *(A)* Significant TR at baseline-before TLE, with ‘coaptation gap’ marked by arrow. No signs of the flail leaflet. *(B)* Flail septal leaflet of TV marked by arrow in TTE. *(C)* Torrential TR in colour Doppler. *(D)* Flail septal leaflet of TV is marked by an arrow in TEE. *(E)* Tricuspid valve after implantation of three PASCAL Ace devices. *(F)* Maintained TR reduction at follow-up visit. RA—right atrium; RV—right ventricle.

An 86-year-old woman was referred for TLE due to an acute phlegmonous pacemaker pocket infection caused by methicillin-sensitive *Staphylococcus aureus* (MSSA). One month before admission, she underwent an elective pacemaker battery replacement. The initial dual-chamber pacemaker had been implanted 14 years earlier for sick sinus syndrome. Treatment with cloxacillin 2 grams six times per day was started, and the patient underwent TLE in accordance with current guidelines.^5^ The pacemaker system was dissected and removed from the pocket, where purulent discharge was noted. The leads (both passive fixation) were fully extracted using Byrd polypropylene telescopic sheaths (Cook Medical, Bloomington, IN, USA). After releasing the adhesions in the superior vena cava, the ventricular lead was completely removed without advancing the sheaths through the TV. The procedure was technically successful, resulting in the complete removal of leads. However, intra-procedural TEE revealed worsening of TR to the torrential grade (EROA & 2.8 cm2, regurgitant volume & 180 mL, rPISA & 17 mm, vena contracta & 12 mm). Of note, severe coaptation defect of tricuspid leaflets—a qualitative echocardiographic feature of significant TR—was observed prior to TLE (*Figure 2A*, movie  *S1*). However, the post-procedural imaging (*Figure 2B–D*, movie  *S2*, *S3*) showed extensive flail of the septal leaflet due to the papillary muscle avulsion, which was absent before the procedure. The TR worsening was accompanied by the acute enlargement of the right ventricle and right atrium, related to severe volume overload (*Figure 2B–D*, movie  *S3*).

The patient’s clinical condition deteriorated, progressing to NYHA Class IV heart failure with peripheral congestion, requiring escalating doses of intravenous diuretics. After completion of antibiotic therapy and Heart Team evaluation due to high operative risk (TRISCORE 22%, TRIVALVE Score 16%, STS mortality and morbidity 23.7%, Euroscore II 10.3%, Supplementary Table  *S1*.^6–9^), the patient was qualified for transcatheter tricuspid edge-to-edge repair (T-TEER), performed under general anaesthesia three weeks post TLE. Three Pascal Ace devices (Edwards Lifesciences, California, USA) were implanted—two in the anteroseptal commissure and one in the posteroseptal commissure (*Figure 2E*, movie  *S4*). The procedure was successful, uncomplicated, and relatively short (60 min), reducing TR from torrential to moderate. The patient’s condition improved significantly, and she was discharged on post-procedure day eight. Pre-discharge echocardiography confirmed the reduction in TR, and at 6-week follow-up, the patient remained clinically stable with preserved procedural results in echocardiography (*Figure 2F*, movie  *S5*).

## Patient 2

**Figure 3:**
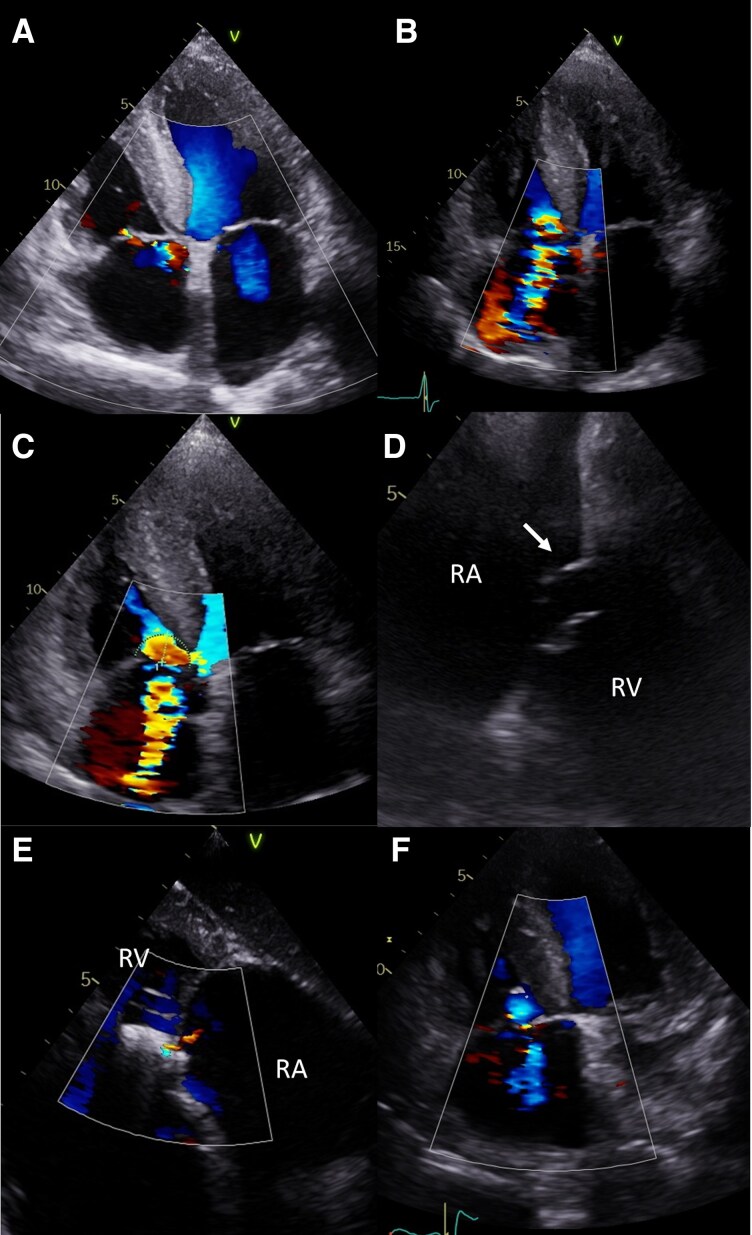
Patient 2 *(A)*. Trace TR before pacemaker implantation. *(B)* Significant TR after pacemaker implantation. *(C)* TR worsening to the massive grade following lead extraction. *(D)* Flail of the septal leaflet of TV marked by arrow. *(E)* Tricuspid valve after implantation of one PASCAL Ace device. *(F)* Maintained TR reduction at follow-up visit. RA—right atrium; RV—right ventricle.

An 83-year-old man with hypertrophic cardiomyopathy and permanent atrial fibrillation underwent a VVI pacemaker implantation four months earlier for high-grade atrioventricular block. Relevant comorbidities included prior endovascular abdominal aortic repair with stent-graft implantation 12 years earlier, re-intervention for an endoleak, moderate aortic stenosis, carotid artery disease, thrombocytopenia, and psoriasis. Baseline echocardiography showed trace TR before the pacemaker implantation (*Figure 3A*, movie  *S6*). However, shortly after the procedure, severe symptomatic TR developed (EROA & 0.7 cm2, regurgitant volume & 51 mL, rPISA: 10 mm) (*Figure 3B*, movie  *S7*). He was hospitalized with NYHA class IV heart failure symptoms, including peripheral oedema and ascites, requiring intravenous diuretics.

To reduce the suspected septal leaflet impingement, the ventricular lead was extracted by simple traction, and a leadless AVEIR pacemaker (Abbott Laboratories, Illinois, USA) was implanted in the same session. Despite this, TR worsened to torrential (EROA & 1.2 cm2, regurgitant volume & 92 mL, rPISA & 15 mm, vena contracta & 9 mm), and the patient’s condition deteriorated further, requiring higher doses of intravenous furosemide. Transoesophageal echocardiography (TEE) revealed a flail septal leaflet in the anteroseptal commissure as the likely cause of the significant TR increase, not observed before TLE (*Figure 3C, D*, movie  *S8*). Given the short time between the pacemaker implantation and TLE it is uncertain when the valve was damaged. It is possible that the chords were torn during the implantation, and the pathology was fully revealed after TLE.

Following Heart Team consultation, the patient was disqualified from tricuspid valve surgery due to the high intervention risk (TRISCORE 48%, TRIVALVE Score 22.6%, STS mortality and morbidity 38.4%, Euroscore II 10.81%, Supplementary Table  *S1*.^6–9^) and underwent successful T-TEER using a single Pascal Ace device. The procedure, performed under general anaesthesia, lasted less than one hour and was uncomplicated. Intra-procedural TEE showed acute TR reduction to mild (*Figure 3E*, movie  *S9*). The patient was discharged home on post-procedure day five. The effect was sustained on follow-up transthoracic echocardiography (TTE) 34 days after the procedure (*Figure 3F*, movie  *S10*).

## Patient 3

A 74-year-old man with a complex cardiac history—including heart failure with mildly reduced ejection fraction (HFmrEF), coronary artery disease (with multiple percutaneous interventions and a coronary artery bypass graft 11 years prior), permanent atrial fibrillation, ventricular septal defect, hypertension, chronic obstructive pulmonary disease, pulmonary hypertension, and type 2 diabetes mellitus—was admitted for the TLE. The dual-chamber pacemaker had been implanted 30 months earlier for sick sinus syndrome.

**Figure 4:**
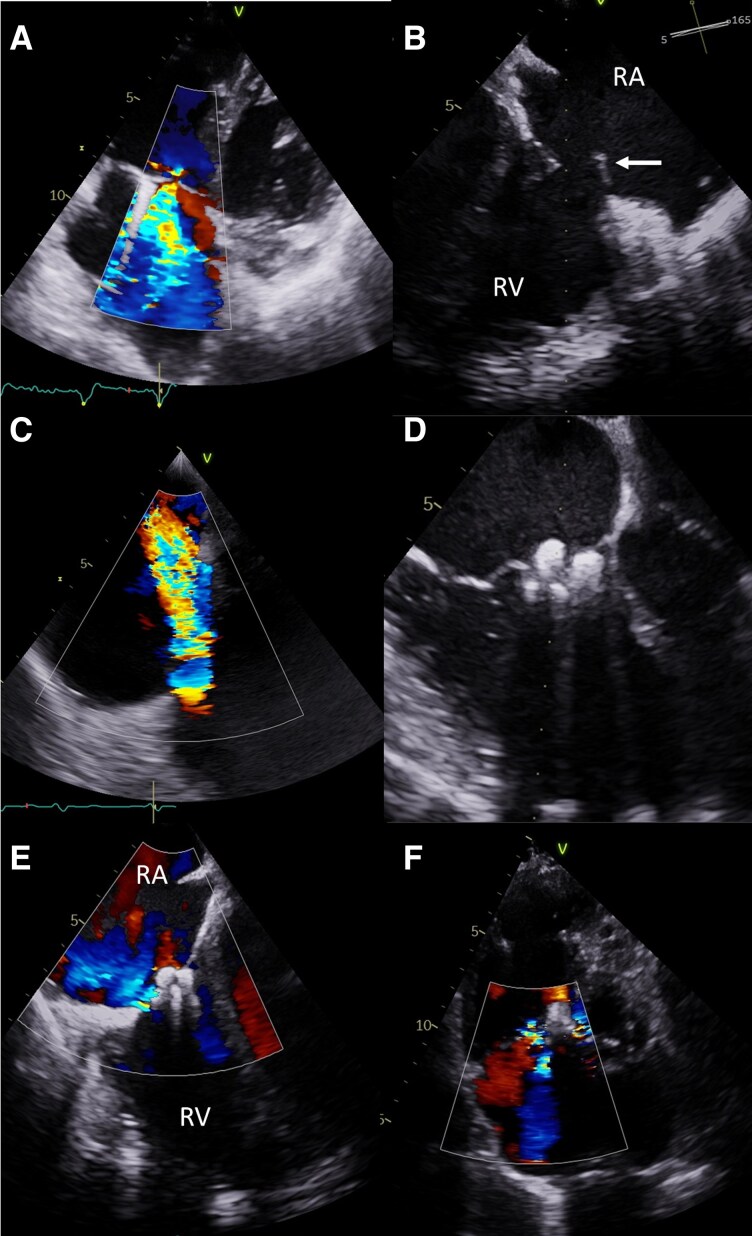
Patient 3 *(A)* Suspected conflict between the ventricular lead and the anterior tricuspid leaflet, causing significant TR. *(B)* Flail of the anterior leaflet of TV marked by the arrow after TLE. *(C)* Intra-procedural echocardiography during TLE documenting progression of TR to torrential grade. *(E)* Tricuspid valve after implantation of two PASCAL Ace devices. *(F)* Reduction of TR to moderate, maintained at follow-up visit. RA—right atrium; RV—right ventricle.

**Figure 5:**
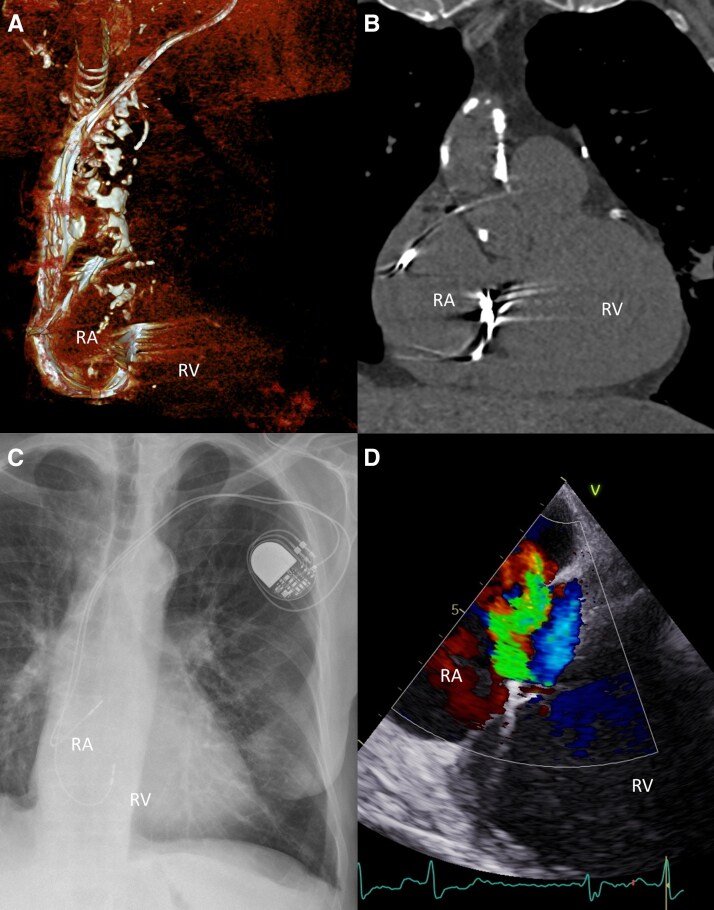
Patient 3. Malpositioned ventricular lead, looped and hypermobile at the tricuspid annulus. *(A)* 3D CT reconstruction. *(B)* CT coronal plane. *(C)* Chest X-ray. *(D)* Transthoracic echocardiography (TTE) showing eccentric regurgitant jet on colour Doppler. RA—right atrium; RV—right ventricle.

The patient was referred for extraction due to a suspected conflict between the malfunctioning (stimulation thresholds in the ventricle > 7.5 V/1.5 ms) ventricular lead and the anterior tricuspid leaflet, resulting in severe TR (*Figure 4A*, movie  *S11*) and worsening heart failure symptoms (from NYHA II to III). Transthoracic echocardiography, chest x-ray, and computed tomography confirmed a mispositioned ventricular lead looped and hypermobile in the region of the tricuspid valve ring (*Figure 5A-D*). Both actively fixated leads were completely removed by simple traction, without the use of additional extraction tools. A new pacemaker system was not implanted due to the current lack of indications.

However, intra-procedural TEE revealed increase in TR severity to torrential (ERO & 1.4 cm2, regurgitation volume & 122 mL, rPISA & 14 mm, vena contracta & 11 mm), with anterior leaflet prolapse and rupture of chordae tendineae (*Figure 4B,C*, movie  *S12*,13). Despite this, the patient remained clinically stable and was discharged on post-procedure day 3 without worsening of heart failure symptoms.

Six months later, the patient developed heart failure symptoms staged as NYHA III and after the Heart Team discussion was disqualified from tricuspid valve surgery due to the high risk (TRISCORE 34%, TRIVALVE Score 40.5%, STS mortality and morbidity 27.1%, Euroscore II 19.83%, Supplementary Table  *S1*.^6–9^) and underwent successful T-TEER with two Pascal Ace devices implanted between the anterior and septal leaflets (*Figure 4D*, movie  *S14*). The procedure, conducted under general anaesthesia, lasted 115 min. TEE confirmed TR reduction to moderate (*Figure 4E*). The patient was discharged on day two post-procedure, with improvement in the heart failure symptoms to NYHA class II. The result was maintained at the follow-up visit 31 days after the implantation (*Figure 4F*, movie  *S15*).

## Patient 4

**Figure 6:**
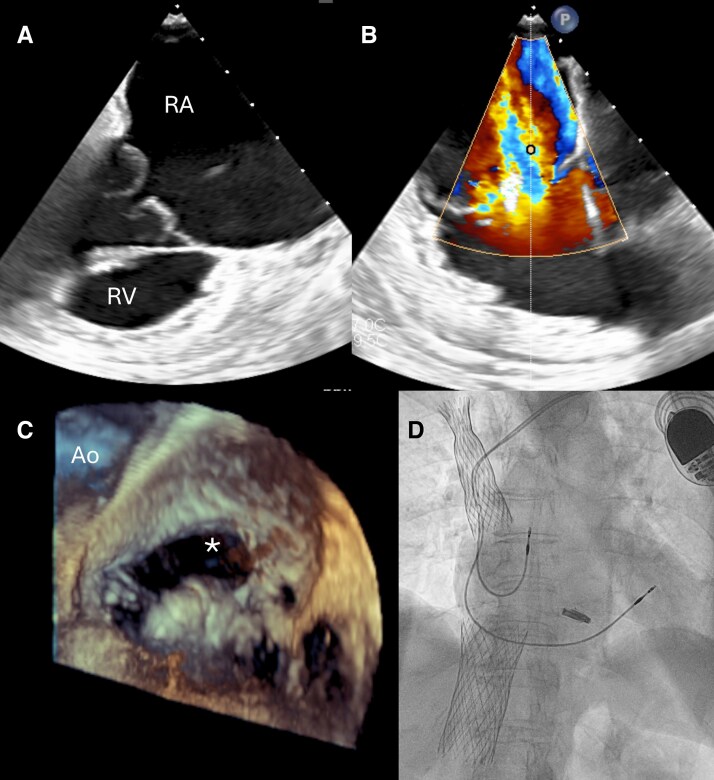
Patient 4. *(A)* Tricuspid valve before device implantation. *(B)* Persistent TR following PASCAL Ace implantation. *(C)* Anterior leaflet perforation (*) detected post-implantation., *(D)* TricValve implantation. Ao—aortic valve; RA—right atrium; RV—right ventricle.

An 80-year-old woman underwent TLE 18 months before the qualification for tricuspid valve repair. TLE was performed due to damage to the ventricular lead, resulting in pacing dysfunction, and was followed by the implantation of a new dual-chamber pacemaker due to high-grade atrioventricular block. The leads were extracted using Byrd polypropylene telescopic sheaths (Cook Medical, Bloomington, IN, USA). The TLE procedure was complicated by the deep vein thrombosis of the left arm and significant TR worsening. In spite of medical treatment with high doses of diuretics, the patient complained of easy fatigue and dyspnoea on mild exertion, with moderate ankle oedema with no signs of ascites. Transthoracic echocardiography showed torrential TR (ERO 1.1 cm2 ; TR Vol 76 mL) and preserved overall right ventricular contractile function. Transoesophageal examination confirmed the presence of torrential tricuspid regurgitant jet (TR vena cotracta area 1,3 cm2) originating mainly from the central region of the valve and anteroseptal coaptation line with 7–8 mm coaptation gap. Possible rupture of secondary chordae to the anterior leaflet was also noted and identified as the predominant cause of regurgitation (*Figure 6A*). During the Heart Team discussion, the patient, due to high risk of surgery (TRISCORE 22%; TRIVALVE Score 16%; STS mortality and morbidity 22.6%; Euroscore II 4,32%, Supplementary Table  *S1*.^6–9^) was qualified for T-TEER. Pascal Ace device (Edwards LifeSciences California, USA) was implanted between the septal and the anterior leaflet in the central region of the tricuspid valve (*Figure 6B*). It led to the reduction of the tricuspid anulus size and changed the distribution of the main regurgitant jet, which moved towards the anteroseptal commissure. Implantation of a second device was considered, further echocardiographic evaluation revealed the presence of a very short part of the anterior leaflet in the commissural region, which was most likely caused by an anterior leaflet perforation during the TLE (*Figure 6C*, movie  *S16*). The procedure was aborted without a significant reduction of tricuspid regurgitation. Given the lack of efficacy of T-TEER and the persistence of clinical symptoms, the patient ultimately underwent bicaval valve implantation using the TricValve system (P&F Products & Features, Vienna, Austria) (*Figure 6D*).

## Discussion

The worsening of primary severe TR is a rare complication of the TLE procedure that occurs in up to 2.5% of cases.^3^ However, when torrential TR complicates TLE, the clinical consequences might be severe as the patient may develop rapidly progressing heart failure with limited response to medical treatment.^4^ This was also true for the patients reported in the current manuscript. Until recently, surgical TR repair was the only option for those subjects. However, the risk of tricuspid valve surgery is often prohibitive in elderly patients with multiple comorbidities, which was confirmed by recent report from which TRI-SCORE calculator was derived.^6^ All patients from our series were elderly and had numerous comorbidities that rendered them unsuitable for surgery. Currently, the T-TEER is the most widely used alternative approach in high-risk subjects with symptomatic severe TR, provided the valve anatomy is suitable, and the TEE imaging quality is good. The flail tricuspid leaflet due to the rupture of chordae tendineae is not seen very often, and the vast majority of TR patients have a secondary aetiology.^10^ However, the T-TEER results for flail leaflets in patients with primary TR are satisfactory.^10^ Our report extends these findings to include iatrogenic injury causing a flail leaflet.

Papillary muscle rupture resulting in acute mitral regurgitation is a well-known complication of myocardial infarction that can be treated with mitral TEER procedure.^11^ Belanger *et al*. described iatrogenic TLE-related papillary muscle rupture supporting the mitral valve in a 33-year-old patient with dextrotransposition of the great arteries corrected with a Mustard procedure in childhood.^12^ Although complex, the procedure described by Belanger *et al*. was done on the anatomical mitral valve. Recently, Golińska-Grzybała K *et al*. reported a case describing iatrogenic torrential TR after TLE of a damaged RV electrode in the mechanism of papillary muscle rupture.^13^ In that case, three MitraClip XTW devices (Abbott Vascular, Menlo Park, California, USA) were used with successful TR reduction to mild-to-moderate.

To minimize the risk of tricuspid valve damage during transvenous lead extraction, controlled, non-excessive traction combined with gentle advancement of a mechanical or dilator sheath should be employed rather than forceful pulling. Intra-procedural TEE and haemodynamic surveillance can further aid early detection of leaflet or chordal injury. This approach is particularly important in patients with long lead dwell times or dense fibrotic adhesions, which are recognized risk factors for worsening tricuspid regurgitation post-extraction.^14^ In the first case, excessive traction due to venous adhesions and interaction of the ventricular lead with the septal leaflet, combined with the patient’s tissue fragility, likely caused mechanical tricuspid valve damage. In the second and third cases, there were no venous adhesions, and the leads were removed with simple traction.

One of the most important factors leading to a T-TEER procedural success is accurate assessment of the tricuspid regurgitation mechanism. In the fourth case, identification of the underlying regurgitation cause was limited by the image quality in transoesophageal examination. It led to the underestimation of the TLE-associated leaflet damage and led to the failure of the T-TEER procedure.

## Conclusions

Percutaneous repair of TLE-related TR is feasible and might be a valuable therapeutic option in patients with prohibitive surgical risk.

## Supplementary Material

ytag164_Supplementary_Data

## Data Availability

The data underlying this article are available in the article and in its online Supplementary material.

## Lead author biography

Jarosław Skowroński, MD, PhD, is an invasive cardiologist at the Department of Interventional Cardiology and Angiology, National Institute of Cardiology in Warsaw, Poland. His clinical focus includes transcatheter mitral and tricuspid valve repair, percutaneous left atrial appendage closure, and high-risk percutaneous coronary interventions. He obtained his PhD in 2020 and was awarded the Prime Minister's Prize for an outstanding doctoral dissertation. Has authored 29 publications, served on European Association of Percutaneous Cardiovascular Interventions (EAPCI) committees, and is currently a co-investigator in the STOP CLOT trial.

## Notes

**Consent:** Written informed consent was obtained from three of the four patients included in the case series, in accordance with COPE (Committee on Publication Ethics) guidelines. These patients were made aware of the implications of publication.

One patient is deceased due to causes unrelated to the subject of the case series. The authors contacted the patient's next of kin, who were informed of the planned publication and gave their written consent for publication.
