# Supplementary material for: The edge-to-edge repair of the iatrogenic torrential tricuspid regurgitation complicating transvenous pacemaker lead extraction: a case series
Source: Eur Heart J Case Rep. 2026 Mar 6;10(3):ytag164. doi: 10.1093/ehjcr/ytag164 (PMC13008292; doi:10.1093/ehjcr/ytag164)
Supplement: ytag164_Supplementary_Data [file ytag164_supplementary_data.zip › Supplementary Table 1 09.12.2025.docx]

Supplementary Table 1. Patients operative and interventional scores risk.

| Patient no. | TRISCORE (5) | TRIVALVE Score (6) | STS mortality & morbidity (7) | Euroscore II (8) |
| --- | --- | --- | --- | --- |
| 1 | 6/12- 22% | 2 – 16% | 23.7% | 10.3% |
| 2 | 8/12 -48% | 2.5-22.6% | 38.4% | 10.81% |
| 3 | 7/12- 34% | 3.5-40.5% | 27.1% | 19.83% |
| 4 | 6/12- 22% | 2 – 16% | 22.6% | 4.32% |
